# Supplementary material for: A complex interplay of various intracellular motifs determines G protein binding and activation of muscarinic receptors
Source: Sci Rep. 2026 Apr 14;16:12370. doi: 10.1038/s41598-026-48667-0 (PMC13083859; doi:10.1038/s41598-026-48667-0)
Supplement: Supplementary file 1 — Supplementary Material 1 [file 41598_2026_48667_MOESM1_ESM.pdf]

Supplemental Information

Table 1: Chimeric Receptor Constructs

Construct	Position removed from WT Receptor	Position inserted from other receptor
M3 ICL2 M2	R177-A178	P312-V133
M3 ICL3 M2	E274 - T471	D299 - K367
M3 TM56 M2	Y251-E274 & T471-A495	Y206-D299 & K367-A391
M3 TM56 + H8 M2	Y251-E274 & T471-A495 & K549- D564	Y206-D299 & K367-A391 & A445- Y459
M3 TM56 + PBC M2	Y251-E274 & T471-A495 & K565-K570	Y206-D299 & K367-A391 & K460-R466
M3 ICL3 + TM56 M2	Y251- A495	Y206-A391
M3 ICL3+ TM56 + PBC M2	Y251- A495 & K565-K570	Y206-A391 & K460-R466
M3 H8 M2	K549-D564	A445-Y459
M3 H8 + CTerm M2	K549-L590	A445-A466
M3 PBC M2	K565-K570	K460-R466
M3 PBC short	K565- L590	K460-R466
M3 -distal Cterm	Q571-L590	-
M3 ICL2 + ICL3 + TM56 M2	R177-A178 & Y251- A495	P312-V133 & Y206-A391
M3 complete M2	R177-A178 & Y251- A495 & K549-L590	P312-V133 & Y206-A391 & A445-A466
M2 ICL2 M3	P312-V133	R177-A178
M2 ICL3 M3	D299 - K367	E274 - T471
M2 TM56 M3	Y206-D299 & K367-A391	Y251-E274 & T471-A495
M2 TM56 + H8 M3	Y206-D299 & K367-A391 & A445- Y459	Y251-E274 & T471-A495 & K549- D564
M2 TM56 + PBC M3	Y206-D299 & K367-A391 & K460-R466	Y251-E274 & T471-A495 & K565-K570
M2 ICL3 + TM56 M3	Y206-A391	Y251- A495
M2 ICL3+ TM56 + PBC M3	Y206-A391 & K460-R466	Y251- A495 & K565-K570
M2 H8 M3	A445-Y459	K549-D564
M2 H8 + CTerm M3	A445-A466	K549-L590
M2 ICL3 + TM56 + H8 + Cterm	Y206-A391 & A445-A466	Y251- A495 & K549-L590
M2 PBC M3	K460-R466	K565-K570

M2 +distal Cterm M3	WT	Q571-L590 added
M2 ICL2 + ICL3 + TM56 M3	P312-V133 & Y206-A391	R177-A178 & Y251- A495
M2 complete M3	P312-V133 & Y206-A391 & A445-A466	R177-A178 & Y251- A495 & K549-L590

Table 2: Oligonucleotide Primers

**Construct**	**Primer Sequence 5' → 3'**
mCherry-M3	AAA AAA GAA TTC CTT GTA CAG CTC GTC CAT GCC<br>AAAAAA GAA TTC ATG ACC TTG CAC AAT AAC AGT ACA ACC<br>CGCCCTGAGCTACATCTTCTGCCTGGTATTCGCCATGGTGAGCAAGGGCGAG<br>AGAAGATGTAGCTCAGGGCGATGATCGTCTTCATGGATCCGAGCTCGGTACC<br>GCAGGCCTTGTAGCGGCCGCTCGAG<br>GCAAGGTCATGAATTCCTTGTACAGCTCGTCCATGCC<br>CAAGGAATTCATGACCTTGCACAATAACAGTACAACC<br>GCGGCCGCTACAAGGCCTGCTCGGGTG<br>AGGCCTTGTAGCGGCCGCTCGAGGGGGGGC
M2-mCit	Digestion and Ligation with XbaI and NotI from M3-mCit
mCherry-M2	AAA AAA GAA TTC ACC ATG AAG ACG ATC ATC GCC<br>AAA AAA GAA TTC CTT GTA CAG CTC GTC CAT GCC
PTX-A pcDNA3	Fw: AAAAA AAGCTT ATGGACGATCCTCCCGC<br>Rev: AAAAA TCTAGA ACGAATACGCGATGCTTTC
**M3 Chimeric Receptors**	
M3 ICL2 M2	GGCCGCTCACGTACC**C**AG**T**CAAACGAACAACAAAG
M3 ICL3 M2	BB fw: CTAAGCAGCCTGCAAAAAAGAGGATGTCCCTGGTCAAGGAG<br>BB rev: GGGTCTTGGTTGGCAACAGGCAGGCCAGCAAGCTC<br>Insert fw: CCAAAGAGCTTGCTGGCCTGCCTGTTGCCAACCAAGACC<br>Insert rev: TCCTTGACCAGGGACATCCTCTTTTTTGCAGGCTGCTTAGTCATC
M3 TM56 M2	Same as M2 ICL3 M3, Template ICL3 + TM56 M2
M3 TM56 + H8 M2	Same as M3 H8 M2, Template M3 TM56 M2
M3 TM56 + PBC M2	Same as M3 pbc M2, Template M3 TM56 M2
M3 ICL3 + TM56 M2	BB fw: CCA AGA GGT TTG CTC TGA AGA AGA TGA CTA AGC AGC CTG CAA AAA AG

	BB rev: ACA AAG TTT TCT GTC TCT GCG TCT TGG TTG GCA ACA GGC T<br>Insert fw: AGC CTG TTG CCA ACC AAG ACG CAG AGA CAG AAA ACT TTG TCC ACC<br>Insert rev: GCA GGC TGC TTA GTC ATC TTC TTC AGA GCA AAC CTC TTG GCC
M3 ICL3+ TM56 + PBC M2	Same as M3 pbc M2, Template M3 ICL3 + TM56 M2
M3 H8 M2	Fw: acaccttctcatgtgtcattatAAAAAAAAGAGGCGCAAG<br>Rev: ttaaaggtcttcttgaaggtggcGTTGCACAGAGCATAGCA
M3 H8 + CTerm M2	BB fw: TACGGCAAGCTGACCCTGAAG<br>BB rev: TTGAAGGTGGCGTTGCACAGAGCATAGCACACG<br>Insert fw: CTGTGCAACGCCACCTTCAAGAAGACCTTTAAACAC<br>Insert rev: CTTCAGGGTCAGCTTGCCGTAG
M3 PBC M2	Fw: gcgctacaaggCAGCAGTACCAGCAG<br>Rev: ctatgttcttGTCACACTGGCACAG
M3 PBC short	Fw: TCTAGAGTGAGCAAGGGC rev: CCTTGTAGCGCCTATGTTC
M3 -distal Cterm	Fw: TCTAGAGTGAGCAAGGGC rev: CTTGCGCCTCTTTTTTTTG
M3 ICL2 + ICL3 + TM56 M2	Same as M3 ICL2 M2, Template M3 ICL3 + TM56 M2
M3 complete M2	Template M2 ICL2 + ICL3 + TM56 M2<br>Insert Fw: TGTGCTATGCTCTGTGCAACGCCACCTTCAAGAAGACCTTTAAACAC<br>Insert rev mCit: TCGCCCTTGCTCACTCTAGACCTTGTAGCGCCTATGTTCTTATAATGACAC<br>Insert rev mCherry: CCCCCTCGAGCGGCCGCTACCTTGTAGCGCCTATGTTCTTATAATGACAC<br>BB Rev: AAGGTCTTCTTGAAGGTGGCGTTGCACAGAGCATAGCACACG<br>BB fw mCit: AGAACATAGGCGCTACAAGGTCTAGAGTGAGCAAGGGCGA<br>BB fw mCherry: AGAACATAGGCGCTACAAGGTAGCGGCCGCTCGAGG
**M2 Chimeric Receptors**	
M2 ICL2 M3	CAAAACCTCTGACCTACC**G**AG**C**CAAGCGGACCACAAAAATG
M2 ICL3 M3	BB fw: GTCAGATCACTAAGCGGAAAAAGCCTCCTCCTTCCCG<br>BB rev: GCCTCTGTCCCAGAGGCTTGCTCCTTCTTGTCCTTCTTTATCCTG<br>Insert fw: TAAAGAAGGACAAGAAGGAGCAAGCCTCTGGGACAGAG<br>Insert rev: TCCCGGGAAGGAGGAGGCTTTTTCCGCTTAGTGATCTGACTTCTGG
M2 TM56 M3	BB fw: TTG TAG CCC GCA AGA TTG TGA CCA GAA GTC AGA TCA CTA AGC GG<br>BB rev: ACC AGA CTT GGA GAA ACG GGC TCT GTC CCA GAG GCT TGC A<br>Insert fw: TGC AAG CCT CTG GGA CAG AGC CCG TTT CTC CAA GTC TGG TAC A<br>Insert rev: TTA GTG ATC TGA CTT CTG GTC ACA ATC TTG CGG GCT ACA ATA TTC T
M2 TM56 + H8 M3	Same as M2 H8 M3, Template M2 TM56 M3

M2 TM56 + PBC M3	Same as M2 PBC M3, Template M2 TM56 M3
M2 ICL3 + TM56 M3	Insert fw: GTG CGA TTC TGT TGG CTT TCA TCA TCA CTT GG<br>Insert rev: TAG ATC CTC CAG TAT AGC ACA GTC ATG ATG ATC ACT GGC<br>BB fw: TGT GCT ATA CTG GAG GAT CTA TAA GGA AAC TGA AAA GCG<br>BB rev: GAA AGC CAA CAG AAT CGC ACT GAG GGT CTG GG
M2 ICL3+ TM56 + PBC M3	Template: M2 PBC M3<br>BB fw: GCCCAGACCCTCAGTGCGATTCTGTTGGCTTTCATCATCACTTGGG<br>BB rev: CTTATAGATCCTCCAGTATAGCACAGTCATGATGATCACT<br>Insert fw: AGTGATCATCATGACTGTGCTATACTGGAGGATCTATAAGGAAACTGAAAAGCG<br>Insert rev: TGATGATGAAAGCCAACAGAATCGCACTGAGGGTCTGGG
M2 H8 M3	atgctgctgctgtgccagtgtgacAAGAACATAGGCGCTACAAG<br>cttgaaagtggttctgaatgttttATTGCAAAGTGCATAGCAG
M2 H8 + CTerm M3	BB fw: TACGGCAAGCTGACCCTGAAG<br>Insert rev: CTTCAGGGTCAGCTTGCCGTAG
M2 ICL3+TM56 + H8 + Cterm	Same as M2 ICL3+TM56 M3, Template M2 H8 + CTerm
M2 PBC M3	mCit: TGACGGCCGCTCGAG; CTTGCGCCTCTTTTTTTTATAATGACAC<br>mCherry: TCTAGAGTGAGCAAGG; CTTGCGCCTCTTTTTTTTATAATG
M2 +distal Cterm M3	mCherry: fw: tttcacaagcgcgcacccgagcaggccttgTGAGCGGCCGCTCGAGCA<br>rev: aatgaccgactgtctctgctggtactgctgCCTTGTAGCGCCTATGTTCTTATAATGACACATG
M2 ICL2 + ICL3 + TM56 M3	Same as M2 ICL2 M3, Template M2 ICL3 + TM56 M3
M2 complete M3	Template M2 ICL2 + ICL3 + TM56<br>BB fw mCherry: TTTCACAAGCGCGCACCCGAGCAGGCCTTGTGATCGAGGGGGGGCCCTATTC<br>BB fw mCit: AGAACATAGGCGCTACAAGGTCTAGAGTGAGCAAGGGCGAG<br>BB rev: AAAGTGGTTCTGAATGTTTTATTGCAAAGTGCATAGCAGGCA<br>Insert fw: CCTGCTATGCACTTTGCAATAAAACATTCAGAACCACTTTCAAGATGCT<br>Insert rev mCherry: ATAGGGCCCCCCCTCGATCACAAGGCCTGCTCGGGTG<br>Insert rev mCit: TCGCCCTTGCTCACTCTAGACCTTGTAGCGCCTATGTTCTTATAATGACAC

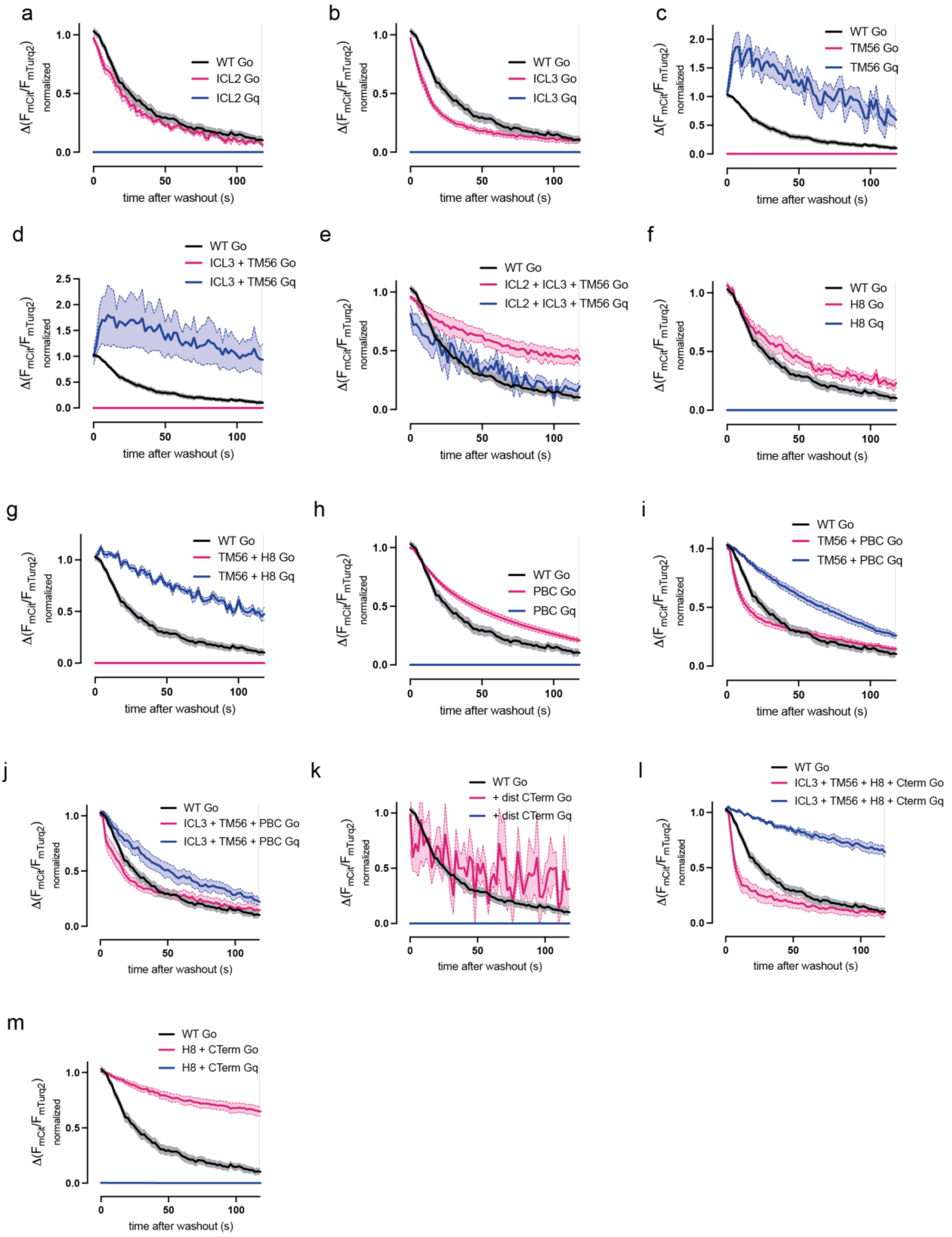

***Supplemental Figure 1: M2-based chimeric receptors enable the binding of Go and Gq proteins by the M2 receptor. a-m** Averaged FRET-based measurement of M2 receptor-G protein interaction in transfected, permeabilized HEK293T cells expressing a mCit-tagged receptor-chimera and the respective G proteins with a Gγ carrying a mTurq with different chimeric*

receptors. Wash-out of the second 10 µM acetylcholine application by buffer solution is depicted (measurement protocol as described in Figure 1c). The area under the curve was calculated to quantify the strength of the interaction and plotted as bar graph In Figure 2 and 4a-b (Mean ± SEM, n=7-13).

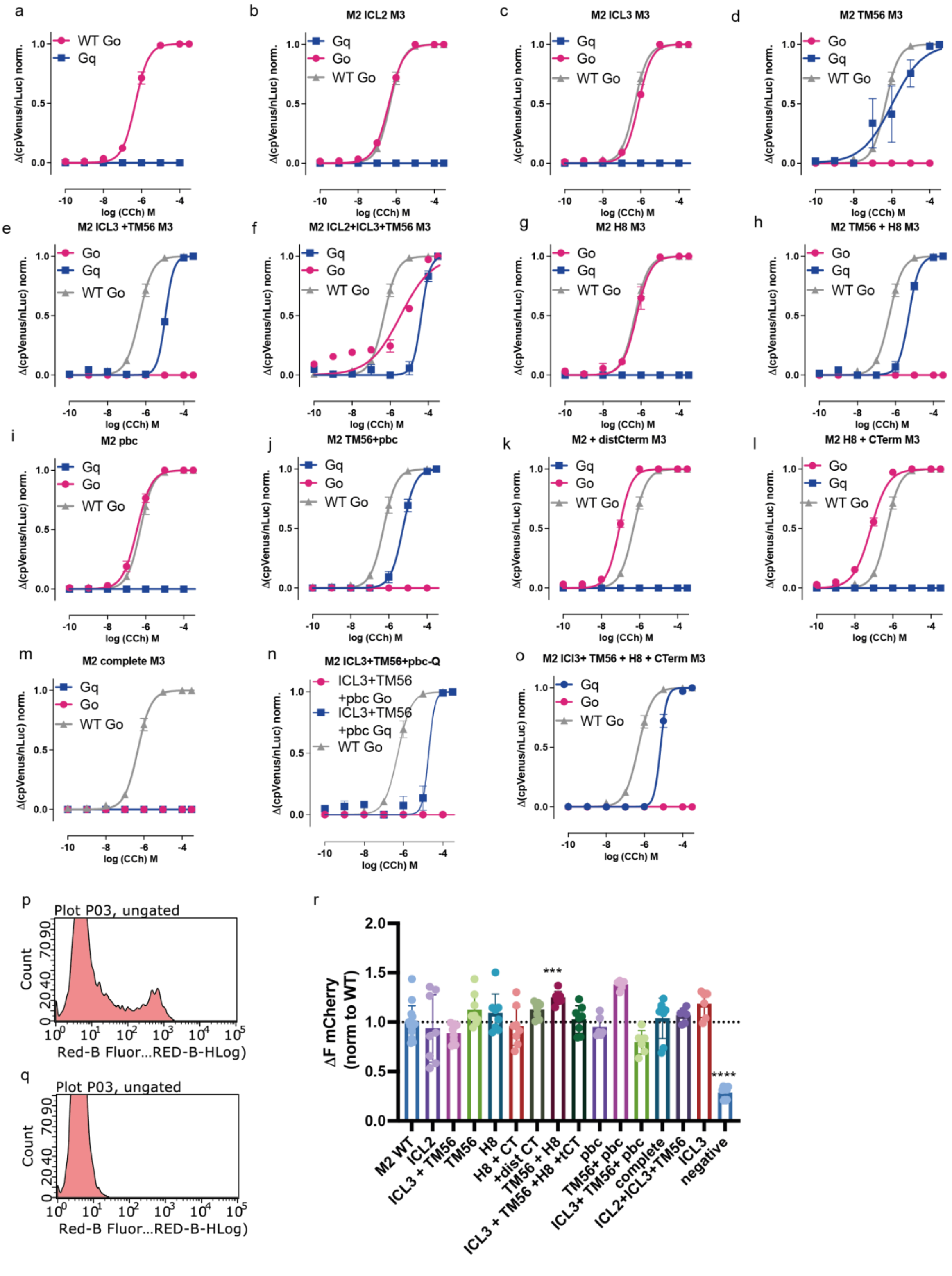

***Supplemental Figure 2: M2-based chimeric receptors enable the subsequent activation of Go Gq proteins by the M2 receptor. a-o** Concentration-response curves for carbachol induced G protein activation, measured using the BRET-based G-case assays in multiwell format in a plate reader. HEK293T cells were transfected with the G protein activity sensor and the chimeric M2-*

based receptor carrying an N-terminal fused mCherry. Concentration-response curves were fitted and the calculated EC50-values were plotted in bar graphs (Figure 2 and 4c-d). Measurements were performed in triplicates for each transfection, with at least three independent transfections. ***p-r*** *Expression control of chimeric M2-based receptors using flow cytometry. HEK293T cells transfected with N-terminal mCherry tagged WT receptor (p), chimeric receptors or no receptor (q) were analyzed via flow cytometry regarding their red fluorescence (p-q). The mean fluorescence was normalized to the fluorescence of the WT receptor and plotted as bar graph (r). Measurements were performed in triplicates for each transfection, with at least three independent transfections. (Statistic: Ordinary One-way ANOVA with Dunnett's multiple comparison test, **** $p<0.0001$, *** $p<0.001$, ns: $p>0.05$).*

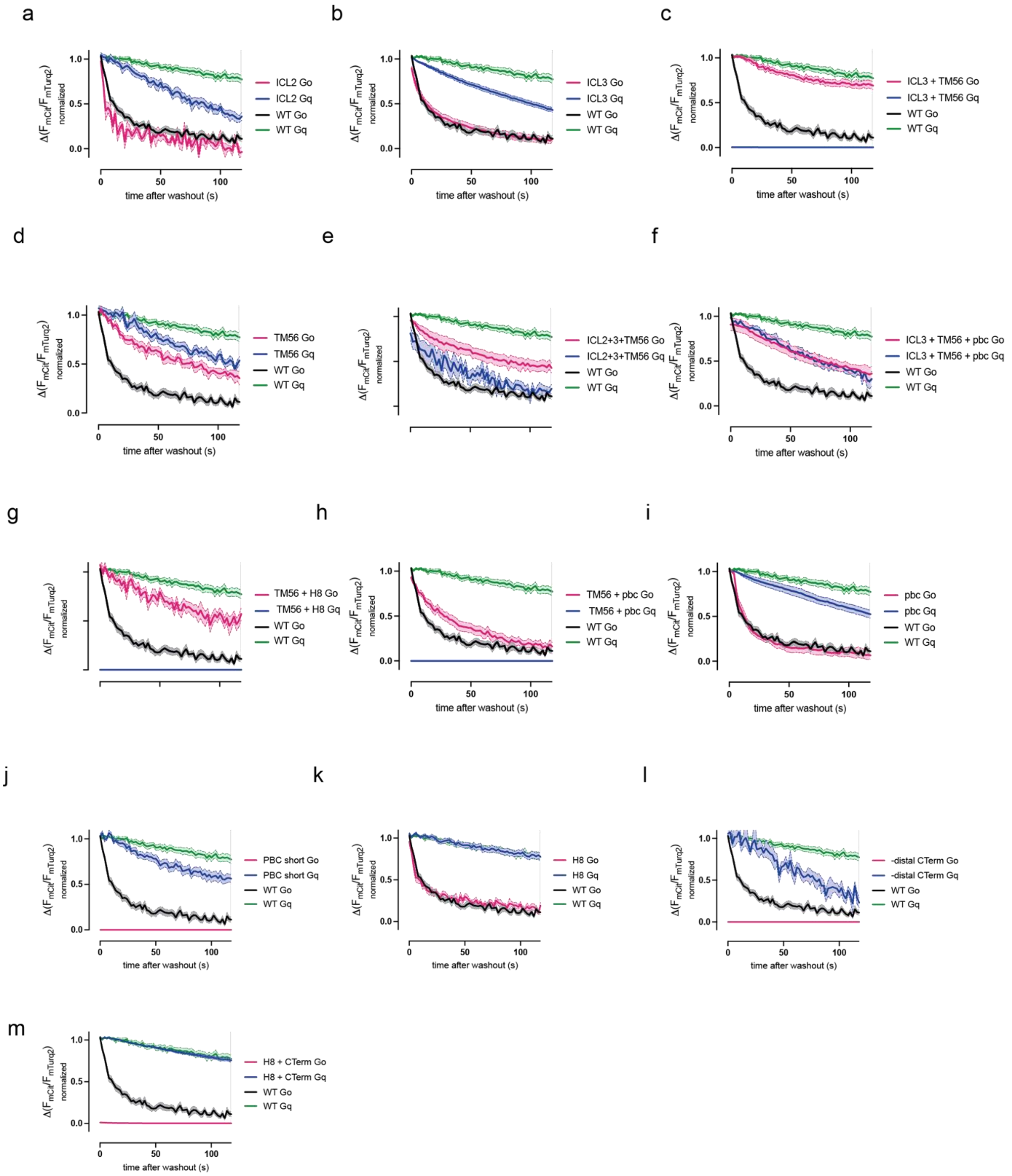

Supplemental Figure 3: M3-based chimeric receptors can be modified to bind solely Gq or Go.

a-m *Averaged FRET-based measurement of M3 receptor-G protein interaction in transfected, permeabilized HEK293T cells expressing a mCit-tagged receptor-chimera and the respective G proteins with a Gγ carrying a mTurq with different chimeric receptors. Wash-out of the second 10 µM acetylcholine application by buffer solution is depicted (measurement protocol as*

described in Figure 1c). The area under the curve was calculated to quantify the strength of the interaction and plotted as bar graph In Figure 3 and 4e-f (Mean ± SEM, n=10-14).

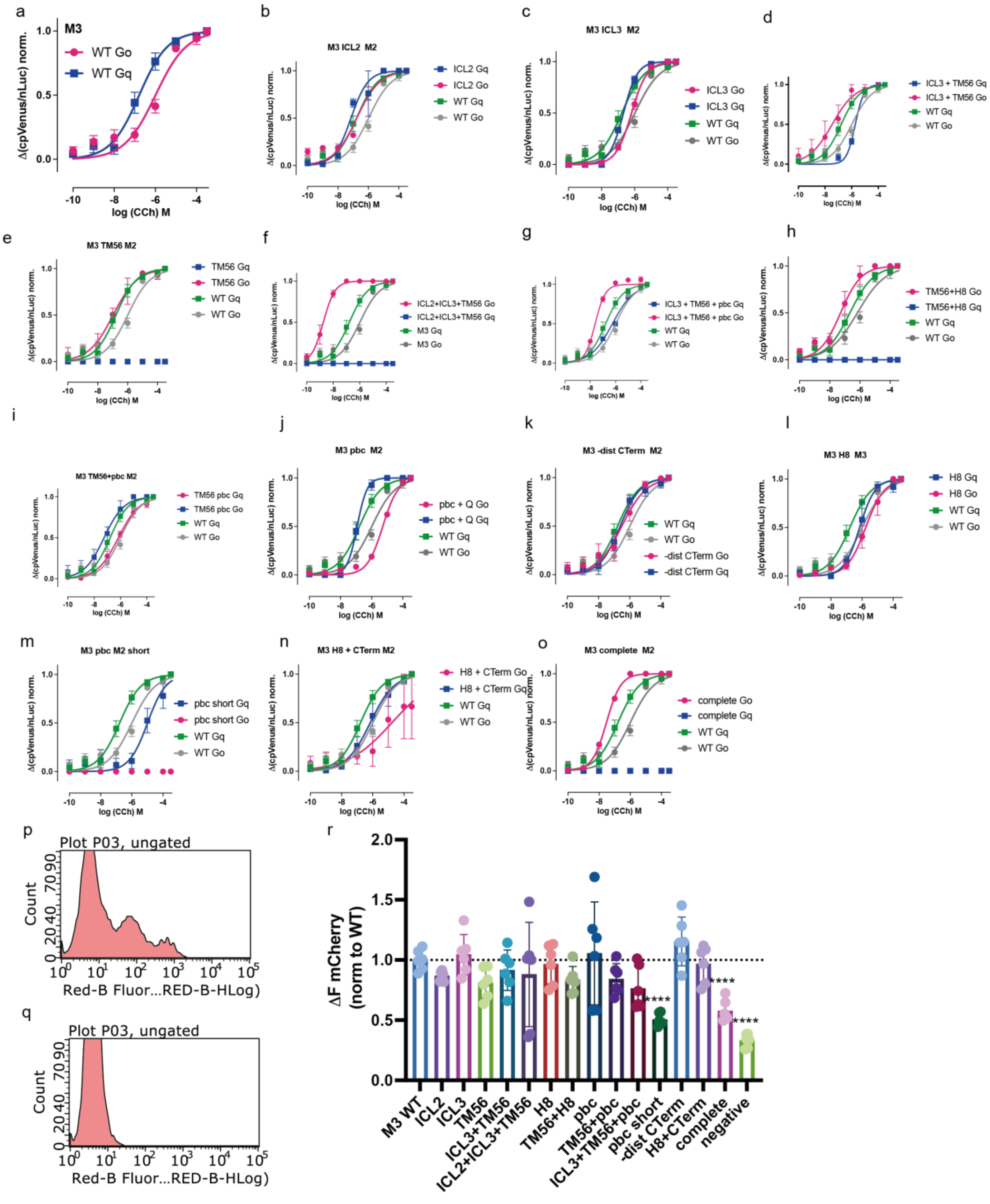

Supplemental Figure 4: M3-based chimeric receptors can be modified to signal solely via Gq or Go. a-o *Concentration-response curves for carbachol induced G protein activation, measured using the BRET-based G-case assays in multiwell format in a plate reader. HEK293T cells were transfected with the G protein activity sensor and the chimeric M3-based receptor carrying an N-terminal fused mCherry. Concentration-response curves were fitted and the calculated EC50-*

values were plotted in bar graphs (Figure 3 and 4g-h). Measurements were performed in triplicate for each transfection, with at least three independent transfections. ***p-r*** *Expression control of chimeric M3-based receptors using flow cytometry. HEK293T cells transfected with N-terminal mCherry tagged WT receptor (p), chimeric receptors or no receptor (q) were analyzed via flow cytometry regarding their red fluorescence (p-q). The mean fluorescence was normalized to the fluorescence of the WT receptor and plotted as bar graph (r). Measurements were performed in triplicates for each transfection, with at least three independent transfections. (Statistic: Ordinary One-way ANOVA with Dunnett's multiple comparison test, **** p<0.0001, ns: p>0.05).*
